# Supplementary material for: Enhancing mitochondrial activity in neurons protects against neurodegeneration in a mouse model of multiple sclerosis
Source: eLife. 2021 Feb 10;10:e61798. doi: 10.7554/eLife.61798 (PMC7993994; doi:10.7554/eLife.61798)
Supplement: Figure 3—source data 1. [file elife-61798-fig3-data1.docx]

**Enhancing mitochondrial activity in neurons protects against neurodegeneration in a mouse model of multiple sclerosis**

**Figure 3 - source data**

| **Time  (minutes)** | **Wild-type** | | | | **Thy1-Ppargc1a** | | | |
| --- | --- | --- | --- | --- | --- | --- | --- | --- |
| 1,303558 | 110,4173 | 94,74146 | 123,9832 | 161,0416 | 153,3209 | 152,5948 | 188,0938 | 233,9225 |
| 7,812006 | 92,44465 | 91,16798 | 115,9044 | 147,5723 | 146,0539 | 142,5883 | 176,1842 | 222,9705 |
| 14,28118 | 84,06895 | 88,70139 | 111,9165 | 142,882 | 142,5059 | 135,9068 | 170,8152 | 217,2791 |
| 20,86343 | 43,36469 | 55,66553 | 67,42881 | 91,53903 | 75,33681 | 60,2016 | 88,9573 | 108,9047 |
| 27,38662 | 42,22688 | 53,14045 | 63,44285 | 81,75394 | 69,27137 | 56,61882 | 82,83165 | 100,9512 |
| 33,82745 | 41,0742 | 50,84478 | 61,08485 | 75,92886 | 68,15402 | 54,98398 | 81,31214 | 99,48068 |
| 40,38946 | 172,8291 | 164,4619 | 210,8062 | 210,9354 | 243,7164 | 237,8295 | 247,9585 | 288,2718 |
| 46,87786 | 165,5542 | 146,3998 | 195,534 | 183,8189 | 218,836 | 221,8465 | 217,4869 | 250,3401 |
| 53,39267 | 156,9148 | 136,7948 | 179,681 | 169,6326 | 202,6081 | 206,192 | 199,112 | 229,1129 |
| 59,95583 | 18,58814 | 36,50655 | 31,44917 | 48,52434 | 42,13562 | 19,21688 | 45,521 | 47,76188 |
| 66,48731 | 19,85293 | 36,29771 | 34,15352 | 48,65565 | 42,49122 | 21,12746 | 46,33419 | 48,24844 |
| 72,97715 | 20,34241 | 36,05096 | 33,0162 | 46,93547 | 43,37259 | 20,72659 | 46,24237 | 47,00735 |
