# Supplementary material for: Enhancing mitochondrial activity in neurons protects against neurodegeneration in a mouse model of multiple sclerosis
Source: eLife. 2021 Feb 10;10:e61798. doi: 10.7554/eLife.61798 (PMC7993994; doi:10.7554/eLife.61798)
Supplement: Transparent reporting form [file elife-61798-transrepform.docx]

***eLife’s* transparent reporting form**

We encourage authors to provide detailed information *within their submission* to facilitate the interpretation and replication of experiments. Authors can upload supporting documentation to indicate the use of appropriate reporting guidelines for health-related research (see [EQUATOR Network](http://www.equator-network.org/%20)), life science research (see the [BioSharing Information Resource](https://biosharing.org/)), or the [ARRIVE guidelines](http://www.plosbiology.org/article/info:doi/10.1371/journal.pbio.1000412) for reporting work involving animal research. Where applicable, authors should refer to any relevant reporting standards documents in this form.

If you have any questions, please consult our Journal Policies and/or contact us: [editorial@elifesciences.org](mailto:editorial@elifesciences.org).

**Sample-size estimation**

- You should state whether an appropriate sample size was computed when the study was being designed
- You should state the statistical method of sample size computation and any required assumptions
- If no explicit power analysis was used, you should describe how you decided what sample (replicate) size (number) to use

Please outline where this information can be found within the submission (e.g., sections or figure legends), or explain why this information doesn’t apply to your submission:

No statistical methods were used to predetermine sample sizes; our sample sizes are similar to those reported in previous publications. This information is stated in the Methods section *Statistics*.

**Replicates**

- You should report how often each experiment was performed
- You should include a definition of biological versus technical replication
- The data obtained should be provided and sufficient information should be provided to indicate the number of independent biological and/or technical replicates
- If you encountered any outliers, you should describe how these were handled
- Criteria for exclusion/inclusion of data should be clearly stated
- High-throughput sequence data should be uploaded before submission, with a private link for reviewers provided (these are available from both GEO and ArrayExpress)

Please outline where this information can be found within the submission (e.g., sections or figure legends), or explain why this information doesn’t apply to your submission:

The numbers of biological and technical replicates are reported in the figure legend of each figure. Biological replicates are n of mice, technical replicates are mentioned as cells. Handling of outliers is described at the end of the Methods section *Statistics*. Criteria for exclusion/inclusion of EAE mice are stated in the Methods section *EAE induction*. High throughput-sequence data were obtained from Schattling B, et al. Nat Neurosci 2019;22:887–896. doi:10.1038/s41593-019-0385-4 under the Gene Expression Omnibus accession number GSE104899.

**Statistical reporting**

- Statistical analysis methods should be described and justified
- Raw data should be presented in figures whenever informative to do so (typically when N per group is less than 10)
- For each experiment, you should identify the statistical tests used, exact values of N, definitions of center, methods of multiple test correction, and dispersion and precision measures (e.g., mean, median, SD, SEM, confidence intervals; and, for the major substantive results, a measure of effect size (e.g., Pearson's r, Cohen's d)
- Report exact p-values wherever possible alongside the summary statistics and 95% confidence intervals. These should be reported for all key questions and not only when the p-value is less than 0.05.

Please outline where this information can be found within the submission (e.g., sections or figure legends), or explain why this information doesn’t apply to your submission:

For all ordinal data (e.g. EAE Score) nonparametric statistics were performed. Interval and ratio data were analyzed for normality and statistical tests were used accordingly. This is stated in the Methods section *Statistics*. Details of the statistical test used for each experiment and sample sizes are mentioned in the corresponding Figure legend. Raw data are presented directly in the bar graphs as scatter plots.

(For large datasets, or papers with a very large number of statistical tests, you may upload a single table file with tests, Ns, etc., with reference to sections in the manuscript.)

**Group allocation**

- Indicate how samples were allocated into experimental groups (in the case of clinical studies, please specify allocation to treatment method); if randomization was used, please also state if restricted randomization was applied
- Indicate if masking was used during group allocation, data collection and/or data analysis

Please outline where this information can be found within the submission (e.g., sections or figure legends), or explain why this information doesn’t apply to your submission:

Wild-type mice were randomized to EAE or healthy group by cages. This is stated in the Method section *Mice*. All other experiments with comparison of transgenic versus wild type mice did not necessitate group allocation methods. For all experiments, the experimenters were blinded to the genotype until the end of the experiment, including data analysis.

**Additional data files (“source data”)**

- We encourage you to upload relevant additional data files, such as numerical data that are represented as a graph in a figure, or as a summary table
- Where provided, these should be in the most useful format, and they can be uploaded as “Source data” files linked to a main figure or table
- Include model definition files including the full list of parameters used
- Include code used for data analysis (e.g., R, MatLab)
- Avoid stating that data files are “available upon request”

Please indicate the figures or tables for which source data files have been provided:

All current data have been provided. Supplemental figures are included in the article file. All individual data are reported within the figure graphs as scatter plots. Numerical data for Figure 3E, 5A and 5C can be found as *Source Data*.

Enrichment analysis was performed using the function ‘gseGO’ of the R package clusterProfiler; plotting was performed with the R packages ggplot2, clusterProfiler and tidyheatmap.

Normalization, calcium spikes, base line detection, and analysis of the calcium clearance rate were performed with the custom-made script written on Python 3.6 (https://github.com/scriptcalcium/PGC1alpha).
